# Book Reviews

**Published:** 1801-04

**Authors:** 


### CRITICAL RETROSPECT

OF

### MEDICAL AND PHYSICAL LITERATURE.

[ FOREIGN AND DOMESTIC. ]

---

*Elementarlehre;* i. e. Elementary Doctrine of Organic Nature, by Dr. F. J. Schelver, Vol. I. Organomy, 1800, pp. 103, in 8vo. *Gottingen, Dieterich.*

It is very consoling to the rational physician, to contemplate the active spirit and the vigorous efforts of the present age, in order to penetrate into the mystery of the final cause of organic phenomena, to point out the constant laws which rule their eternal vicissitude, and by that means to establish a higher degree of certitude and solidity in the medical art than it has hitherto enjoyed. Every contribution tending to that purpose, ought to be thankfully received, and every idea duly regarded, by which we may hope to advance in the intricate knowledge of our organization. The ingenious author of this publication has endeavoured to contribute his share towards its attainment, and by applying the principles of philosophy, to throw new light upon the elements of physiology; in which attempt we readily grant, he has not been unsuccessful, and has not only shown a great deal of acuteness and penetration, but also brought forth several interesting ideas, which, if farther prosecuted, are likely to bring us nearer to truth, and to improve our knowledge of organic nature. After having shortly explained in *the introduction,* the purpose of his work, the author divides the first part of it, or the *organomy,* in which the general as well as particular laws of organic nature are investigated, in the subsequent sections. 1. *On the nisus formativus of nature.* Here the author expounds the idea of *matter,* according to Kant's principles, by adopting only two fundamental powers, the *attractive* and *repulsive,* from which the existence of *matter* is deduced. 2. *On the ideas of organic and inorganic nature.* The author here very justly draws the following conclusion: "Inorganic is quite a relative idea, and nature is an organic whole." 3. *Difference of life and organization,* in which however he seems not to distinguish accurately enough, as it would be proper previously to fix the notion of life before that difference should be stated. 4. *On vis vitalis.* The definition which he gives of vis vitalis, appears to be very plausible. "Life, he says, is the combination of proportionate powers, which by their reciprocal action, produce the phenomena which we call life." 5. *The living organic body more closely contemplated.* This is a recapitulation of his former statements, and an account of the laws of the vis vitalis. 6.

*Difference between Animals and Vegetables.* After having examined the different definitions, by which the naturalifts attempted to conftitute the difference between the animal and vegetable creation, the author adopts the opinion of Prof. FICHTE, who thus ftates it. "Plants are formed and nourifhed by raw matter, whereas the animals receive their nourifhment from the regnum organifatum." 7. *On the difference between man and animal.* This fection is merely metaphyfical, and the refult of it is, "In men there is an organization for *freedom*; in the other organic bodies, there is an organization for *inftinct*." 8. *Elements of the phyfiology of the living bodies.* The author divides the whole fyftem of phyfiology into the vegetable, animal, and human nature, viz. I. ORGANIZATION FOR THE INSTINCT, a, *nutrition by inorganic matter* (plant) b; *nutrition by organic matter,* (animal.) I. ORGANIZATION FOR FREEDOM, (man.) 9. *On the fcope of nature;* very metaphyfical. 10. *On the fcope of living bodies;* where he fays, that all fhall reciprocally nourifh, preferve, and form themfelves. 11. *Deduction of the organic body;* a recapitulation of all its different laws and conditions: This fection is written with particular acutenefs.

---

*Olai Swartz,* difpofitio fyftematica mufcorum frondoforum Sueciæ. Adjectis defcriptionibus et iconibus novarum fpecierum. Erlangæ, apud J. J. Palm. 1799. p. 112 in 8vo. minor. Tabb. Cen. IX.

THIS publication ought to be a very acceptable prefent to the connoiffeurs and lovers of the finer Botany, as it exhibits a defcription of the numerous moffes of Sweden, a country fo rich in cryptogamical plants, and is at the fame time compofed by a botanift, who is known to be particularly converfant in the difficult tribe of moffes. This part of the Swedifh Flora has received confiderable improvements, from the affiduity of Prof. SWARTZ, fince LINNÆUS'S time, as of the two-hundred and three fpecies which are here enumerated, ninety-eight were unknown to LINNÆUS. We have however much reafon to fuppofe, from the extent of that kingdom, and its variety of foil, that there may be ftill feveral fpecies, which efcaped the notice of our author, viz. *Orthotricum Offine, Grimmia Cribrofa, &c.* In the arrangement of the genera, he follows the method of HEDWIG, according to the alterations and improvements that it has undergone by Dr. SCHREBER, from whom however he differs in fome refpects; though this feems not always to merit the approbation of botanifts. Each fpecies is well characterifed, a few inaccuracies and miftakes excepted; and with refpect to the fynonymous terms, he has moftly confined himfelf to DILLENIUS, LINNAEUS, and HEDWIG. Several trivial names are altered, becaufe they gave rife to confufion and miftakes, and feveral doubts are removed, to which fome fpecies of LINNAEUS and DICKSON were expofed. The external neatnefs and elegance of the book keep pace with its internal value. The plates are well executed and neatly coloured, reprefenting moftly new fpecies, as *Grimmia alpicola, Didymodon cernuum, Dicranum*

*cranum Schreberi, Orthotrichum obtusifolium et pumilum, Polytrichum, longisetum, Arcticum, &c.* We regret that from the plan of our Journal we cannot give a fuller account of this work, adequate to its merits, however, we hope that it will come into the hands of every botanift who ftudies the cryptogamical plants.

---

*Europens Vorzugliche Bedurfnisse;* i. e. The principal Neceffaries of Europe from Foreign Countries, Botanically and Chemically examined and confidered with refpect to their Dietetic and Medical Ufe; by Dr. C. W. Juch, &c. No. I. *Coffee and its Substitutes.* 1800; pp. 720, in 8vo. Nurnberg.

Dr. Juch, whofe name is already known to our readers, propofes in this publication to examine fcientifically the different productions that are imported from foreign countries, and the ufe of which has become at the fame time univerfal and neceffary through all Europe, from the higheft ranks to the loweft clafs of people, as *coffee, tea, &c.* and likewife to inveftigate their medical and dietetical qualities, and how far the different fubftitutes anfwer their purpofe. The prefent number is devoted to coffee and its fubftitutes, a beverage particularly familiar upon the continent. After having premifed the botanical defcription, the author proceeds to give a chemical analyfis of coffee and its fubftitutes, according to his own experiments. He analyfed the coffee of Martinique, and found by diftillation, and the preparation of the watery extract, and treating the refiduum with fpiritus vini, in 300 grains, 80 grains of extractive matter, (in which, neither tanin nor an acid could be traced); in 200 grains of the refiduum, 18 ½ grains of a pure bitter refin; 20 grains of the laft refiduum, when treated with nitrous acid, yielded a fubftance like wax, that did not fuffer any change by repeated edulcorations; with potafh a faponaceous mixture was obtained. (Here the author remarks, that common wax is compofed by the carbon and hydrogen of the pollen of flowers, the azote of the bee, and the oxygen of the atmofphere.) At the burning or roafting of the coffee beans, carbon is difengaged, and by uniting with hydrogen forms an oily fubftance, with which the refinous particles combine themfelves. The oxygen of the atmofphere feems alfo to be active, as by roafting the coffee too much, acetous acid is evolved. The goodnefs of the coffee depends on the proportion of the quantity of refin to the extractive matter, on which account bad coffee may be improved by pouring hot water upon the beans, over which it is fuffered to ftand for fome time, which procefs deprives them of a part of extractive matter. 1000 grains of fuccory root, (*Cichorium Intybus, Lin.*) a fubftitute that i moft generally in ufe, yielded 250 grains of a very bitter watery extract, and 30 grains of refinous extract. The root of *Scorzonera Italica, L.* another fubftitute for coffee, afforded of 100 parts, 0,32 water, 0,10 fweetifh watery extract, 0,09 farinaceous matter, 0,03 refin, 0,46 fibrous fubftance. The beet root contained 0,47 water,

 0,13

0,13 ſweet watery extract, 0,07 ſugar, 0,04 albumen, 0,04 ſal ammoniac. This root affords the beſt ſubſtitute. After having previouſly extracted the ſaccharine particles, it ought to be carefully dried, and gently roaſted. The medical virtues of coffee are, according to the author, excitant; and he denies that coffee relaxes the fibre, as is by ſome ſuppoſed. He alſo ſtates how far the uſe of coffee is wholeſome or unwholeſome, according to age, conſtitution, climate, &c. He recommends us to drink it one or two hours after the meal, or, which is ſtill better, one hour before it. It agrees better with perſons of leſs irritability than with others, &c. As a remedy, it proves of ſervice in ſcrophula, hypochondriaſis, aſthma, diarrhœas, agues, againſt narcotic poiſons, &c. The ſalubrity of the ſubſtitutes ſeems to depend upon the ſimilarity of their conſtituent particles with the true coffee, and on the mode of preparation. The beet root deſerves to be preferred to all other ſubſtitutes; however, none of them is able to replace the genuine coffee, which is always more agreeable to the taſte. Theſe are the contents of the firſt number of this uſeful publication, and its continuation ſeems very deſirable.

---

*Filicum Genera et Species Recentiori Methodo Accomodatae, Analytice Descriptæ,* a JOANNE HEDWIG, M. D. ac Profeſſore Botanices in Studio Literario Lipſienſi, &c. Iconibus ad Naturam Pictis Illuſtratae a Romano, Adolpho, Filio, Phil. et M. D. 1799. Lipſiae. Six plates, and five ſheets and a half. Price 12s.

THIS is the laſt production from the pen of that great botaniſt, the late Mr. Hedwig; but the firſt Faſciculus we have before us, makes us wiſh that it may be continued after the author's death. After having made ſome remarks on the terms uſed in the deſcription of ferns, on their generic character according to the parts of fructification, the author proceeds to fix the character of the genus *Trichomanes*, to which the preſent faſciculus is devoted. Dr. Smith had ſeparated this genus into two different genera, viz. *Trichomanes* and *Hymenophyllum*, which however Dr. Hedwig thinks not neceſſary; and beſides, as the character of both genera eſtabliſhed by Doctor Smith, appears to him not ſufficiently clear and diſtinct, he unites them again under *Trichomanes* for preventing confuſion. The deſcription of the ſingle ſpecies is done with much accuracy, and the neceſſary ſynonyms are added, together with the place of growth, and the explanation of the plates. The ſpecies deſcribed and figured in this Faſciculus, are *Trichomanes crinitum* from Jamaica, *Tr. pyxidiferum, L. Tr. hymenoides,* a new ſpecies, *Tr. pinnatum,* likewiſe; *Tr. criſpum,* Plum. *Tr. scandens, L. Tr. lucens, Sw.* from Jamaica; *Tr. puſillum, Sw. Tr. rigidum, Sw. Tr. tenellum, Sw. Tr. reptans.* The plants are repreſented in their natural ſize, and alſo magnified; the ſingle parts of fructification are particularly figured with accuracy and elegance.

---

*Aſclepiades*

*Asclepiades*; i. e. Asclepiades and John Brown, a Parallel by Dr. K. F. Burdach. 1800. Leipsic, Meisner, 8vo.

The history of Asclepiades the Bythinian, has been but lately successfully treated. Asclepiades was at the head of the methodic sect, and his name obtained a very high celebrity, equal to that which Brown begins to enjoy as the author of a system of medicine, that meets with so much applause, particularly on the Continent. It is therefore not an unlucky idea of Dr. Burdach, to draw a parallel between these two men and their respective doctrines, which have occasioned such a revolution in the medical science; and the manner in which he has acquitted himself of this task, renders his book very agreeable to read. He commences with representing their individual qualities, situation, character, and scientific knowledge, and explains the circumstances that gave rise to both their systems, of which he gives the elements. The whole is concluded with observations on both systems.

---

*A System of Dissections, explaining the Anatomy of the Human Body, the Manner of displaying the Parts, and their Varieties in Disease. Vol. I. containing the Dissections of the Abdomen, Thorax, Pelvis, Thigh, and Leg*; by Charles Bell, Fellow of the Royal College of Surgeons. Folio, in five numbers, 5s. 6d. each. Johnson, London.

We have delayed our observations on this very useful work, and excellent guide to the student of Anatomy and Surgery, till the completion of the first part of the author's design.

This volume comprehends the dissection of the abdominal muscles and viscera, with the effects of disease on these parts; the anatomy and diseases of the contents of the thorax; diseases of peritoneum; section of the pelvis; points of surgery illustrated by the section of the pelvis; contents of the pelvis as seen from behind, and plan of the arteries; the descent of the testicle; hernia and hydrocele; dissection of the fascia, superficial parts on the fore part of the thigh; diseases of the cutaneous veins, lymphatics and fascia; ligament of the thigh and femoral artery in the groin; tumors in the groin; popliteal aneurism; and dissections of the back part of the thigh, and of the leg and foot.

The drawings from which the engravings are taken, are highly creditable to the author's ingenuity, and furnish the student with a fair example of the utility, we had almost said the absolute necessity, of the art of designing, for those who wish to become good anatomists and operators.

The author gives several useful directions on the mode of making anatomical preparations by injection, which we select for the gratification of those who have not opportunities of consulting the original.

" To those who are commencing their operations, small subjects will be found the most convenient, being more easily managed, and not likely to embarrass the student with much confusion. Besides, his views at first should not be so immediately directed directed to practice; his object should rather be to acquire general ideas of the anatomy. Young subjects are likewise much fitter for injection (I mean for the injection of the arteries, and for minute injection): they are not only more easily heated and managed, but, what is of more consequence, their blood-vessels have an elasticity and strength which enables them to bear the push of the injection better, and, by a kind of elastic resistance, to give warning of the danger of rupturing their coats; while, in old bodies, the piston of the syringe goes easily down so far, stops, and, if forced, most probably bursts the vessels, driving the injection amongst the muscles, and giving much trouble in the dissection. When any of the trunks burst in this way, the tension being taken off, their coats contract upon the warm injection, and they remain half filled.

"In old age, this want of pliancy becomes very remarkable. There is often a kind of stiffness and rigidity, as if the coats of the vessels were corrugated; a degree of that state in which we find the arteries when ossified, or when concretions are formed in their coats.

"If only some coarse injection is in a slovenly manner to be thrown into the great vessels to show their course, it does not much signify how it is done, or what injection is used, or what means are employed to facilitate the passage of the injection. But if the vessels are to be injected minutely, it is necessary previously to heat the subject well, by bathing it in warm water, or applying steam to the surface. This is of more consequence than even the choice of the subject; for, as the injection is intended to be pentrating and fluid when warm, and, upon becoming cold, to congeal and remain solid in the vessels, it is necessary that the vessels be heated, that they may not suddenly chill the injection: besides, this heating of the body softens and relaxes all the mass of flesh, and brings it to a more suitable state for admitting injection. But it ought to be remembered, that, if the parts be overheated, especially where the vessels to be injected lie exposed, there is danger of spoiling all, by corrugating their coats, and making them quite friable and tender. There is a better way still of heating the subject; viz. by heating the vessels themselves; and, indeed, the two methods should be always combined.—The common practice in the injection of the great vessels, is, to inject first equal parts of brown and white spirit varnish, coloured with the same paint that is used for the coarse or wax injection; and this fine varnish injection, being moderately heated, and thrown in before the wax injection, clears its way, and moderately heats the vessels, so that they do not readily cool or retard the wax injection which is to follow. But when using minute injection (which is size coloured with vermilion) for the purpose of demonstrating the minute vessels, although the hard injection is thrown into the vessels after it, simply to stop the regurgitation of the warm and liquid size, and to retain it in the minutest extremities of the vessels, yet it infallibly happens that the wax injection runs more more minutely in this way than in any other. This being the cafe, it will be found, in all cafes, to be a better method to ufe painter's fize, coloured with vermilion, and heated, but not fo much as to crifp the veffels; and to throw it in before the coarfe injection. It is the leaft expenfive, runs more minutely, gives always a chance for beautiful fpecimens of minute injection, and can be pufhed to any quantity, even till the fkin of the limb becomes quite tenfe, without rupturing the veffels, or thofe veffels at leaft by which the coarfe injection can efcape. By this means, the veffels are dilated, the limb made warm and moift, and the wax injection flows eafily into the arteries, whilft the fize efcapes with the flighteft preffure into the cellular texture.

"There are ftill other things which require attention; viz. the tying of all collateral veffels that may have been opened, and the fixing of the tube fecurely in the mouth of the veffel. When the injecting pipe is introduced into the veffel, it cannot be retained there by a fimple knot, without a chance of its flipping off during the injection, or, if tied firmly, of cutting the coats of the veffel. Therefore, after the ligature is drawn upon the artery including the tube, the ends of the ligature fhould be brought over the wings of the tube, and then carried round fo as to include that part of the ligature which reaches from the mouth of the tube to the wing; and being tied there, the former knot is tightened, and the mouth of the artery drawn up upon the barrel of the tube.

"The coarfe injection is compofed of the following ingredients: Bees wax, fix ounces; refin, eight ounces; turpentine varnifh, fix ounces. The wax and refin give hardnefs and confiftency; and the varnifh is added to give it pliancy. Thefe colours are generally ufed: Vermillion, king's yellow, flake white, fmalt, verditer, verdigrife, lamp black. They fhould be mixed with the turpentine varnifh, and then added to the wax when melted; and fhould there be occafion to melt the injection a fecond time, the heat muft be cautioufly applied, left the colours fhould be burnt and deftroyed. The injection fhould not be thrown into the veffels while too warm, for it will hurt their coats. The degree of heat fhould be fuch, that the finger can be allowed to remain in it for a little while.—A coarfer compofition may be made with tallow, wax, fpirit of turpentine, and oil, coloured with the coarfer paints; or, fimply, tallow and red lead, when the parts are not to be preferved. And for minute injection, turpentine, coloured with vermilion, (which Haller preferred to all other injections, for running minutely, and without extravafation); painter's fize, coloured with any of the above paints; or equal parts of brown and white fpirit varnifh.

"When delicate membranes are to be injected either with quickfilver or with fine fize, inftead of tying all the veffels by which the fluid may efcape, I have found it neceffary only to fear the edges of the membrane with a heated iron; or, after having fixed the tubes, the common method is to dry the edges all round, while the middle part is kept foft and moift. When it is required to to demonſtrate the vaſcularity of a part where there is no opportunity of injecting it, if membranous, the blood may be detained in the veſſels by quickly drying and varniſhing it. The blood, when extravaſated, or when (as in the piles) preternaturally collected in veſſels, may be coagulated by a ſolution of alum; or blood inflamed parts may be coagulated by diſtilled vinegar. In other inſtances, as in preparations of the lacteals, their natural fluids may be coagulated and preſerved by plunging them ſuddenly into ſtrong ſpirits.

"There are many parts of the body which it is impoſſible to keep for any time in their original beauty, and theſe the moſt delicate and intereſting; as the organs of the ſenſes, and all minute nervous parts, the villi of the inteſtines, the comparative anatomy of inſects, the incubated egg, &c. The ready demonſtration of ſuch delicate parts in the freſh ſubject is the trueſt teſt of the abilities of the practical anatomiſt; for there is more delicacy and nicety required in expoſing theſe parts, and more real benefit to be derived from them, than in making the more laſting preparations.—The minute ſtructure of many of theſe parts muſt be diſſected and unravelled under water, where the looſe and floating membranes diſplay themſelves; while, out of the water, they would lie collapſed and undiſtinguiſhed. In ſuch inveſtigations, I have found nothing of ſo much ſervice as jelly made ſtrong and quite tranſparent. When a delicate part is completely diſſected (ſuppoſe it to be the coats of the eye,) place it in the jelly as it is becoming firm, and hold out the parts; and they will be retained, elegantly diſplayed, either for demonſtration or for drawing."

The following obſervation is well worth the attention of ſtudents.

"From what I have ſeen of private diſſection, I would rather adviſe thoſe who are deſirous of undertaking a complete courſe of diſſections, not to begin their labours with learning all the muſcles of the body; for this, beſides other diſagreeable circumſtances, is a dry and tedious taſk at firſt.—It will perhaps be found more truly uſeful to begin their diſſections with general views to the economy of ſuch parts as, from lectures or books, they know to be of importance; then proceeding, in a more determined way, to ſtudy rigidly the anatomy of the bones and muſcles, and accidents of the great joints,—the blood-veſſels and nerves, and the anatomy of the great operations of ſurgery.

"During diſſection, there are many little operations which ſhould be practiſed, and which are neglected. The introducing, for example, of probes into the ducts; as into the naſal duct, and into the ducts of the ſalivary glands; the introducing of inſtruments into the noſe and throat, and into the Euſtachian tube: the uſe of the probang, and of the catheter, &c.—Knowledge and dexterity in ſuch points often prove more uſeful, as being oftener required, than the greater operations of ſurgery."

[ To be continued. ]

*Aſthenology.*

*Asthenology, or the Art of preserving feeble Life, and of supporting the Constitution under the Influence of incurable Diseases.* By C. A. Struve, M. D. Tranflated from the German, by W. Johnston, 8vo. pp. 431, price 8s. London. Murray and Highley.

On the interefting fubject of this work, the Author feems to have exhaufted every fource of information, and to have fuffered nothing connected with his fubject to efcape him. On the importance of Afthenology, efpecially at this time, the author in the introduction fays, "Thofe acquainted with the value of human life, know the importance of a year, a day, and even an hour; and thefe when fpent amidft the full enjoyment of the vital functions, of how much importance to our whole exiftence! What events, fertile in confequences, depend often on one hour of our life! It is, therefore, an eternal and irreparable lofs, when not enjoyed as it ought. On the bed of death, an hour often determines the fate of whole families and ftates. How many fick die in greater peace, becaufe, by having lived an hour longer, they accomplifhed one of their moft ardent wifhes. With what anxiety do many dying fathers wifh for fuch an hour, becaufe they expect the arrival of an abfent fon. How grateful is this hour to furviving friends, who have received from a dying man information refpecting fome important event of his life. Is any thing farther neceffary to give importance to the art of prolonging feeble life?

"In the prefent period, when the number of afthenic difeafes is fo confiderable, this art muft recommend itfelf; and indeed, never was it fo neceffary both for the phyfician and the patient. This is the cafe in particular, among phyficians of the firft clafs, who have to ftruggle againft artificial as well as natural debilities. It too often happens that afthenia exifts to fuch a degree among enervated people, weakened by their mode of life, that no hope is left of reftoring their loft health, much lefs of converting their increafing infirmities into ruftic health and ftrength; and that the higheft triumph which the medical art can obtain, is to affift the gouty voluptuary, or the nervous lady of fafhion, to hold out for a couple of years longer; efpecially when he has to counteract or overcome inceffant irregularities and debilitating habits, which his patients will not abandon. How many phyficians, by their fituation, are obliged to acquire experience in this great art, and yet are blamed becaufe the artificial life which they procure to their exhaufted patients, for a number of years, comes at length to an end."

This volume is divided into two parts; the firft is intitled *Asthenogeny*, in which the nature of the vital principle, animal organization, the fymptoms or phænomena of feeble life, and afthenic difeafes, together with their caufes, are minutely explained.

In his concluding obfervations on the vital principle, Dr. S. obferves, "The vital principle may exift in an organized body in very different degrees. This difference depends on the organization of the body itfelf; according as it is more or lefs fufceptible of the vital principle.

"This difference of fufceptibility is obferved,

"1. In the nature and conftruction of different bodies; thus there is more vital principle in plants than in animals.

"2. In the particular vital fufceptibility of organized bodies of the fame genus; thus fome men have a much greater quantity of the vital principle than others.

"This fufceptibility of the vital principle is,

"1. ORIGINAL: innate as it were; *fortes creantur fortibus.*

"2. ACCIDENTAL; it is deftroyed by difeafes which effect a derangement of the organization.

"A total lofs and deprivation of the vital principle, and a commencement of the free action of the chemical powers in the body, is death.

"Death enfues:

"1. In confequence of the organization being deftroyed, by which means it is rendered incapable of being acted upon by the vital principle. This is effected,

"By the force of external powers and violent impreffions:

"By the gradual decay of vital fufceptibility in the organization in the courfe of time: death in old age, when the organs become infenfible to external ftimulants; when their fufceptibility of irritation even is blunted, by which the progrefs of the bodily functions is rendered fluggifh and flower, fo that they can be excited only by uncommon ftimulants, till the machine at length ftops:

"By the long continuance of very violent excitement, or an immoderate activity of the vital principle, which produces weaknefs and death; an irregular life, uninterrrupted mental irritation.

"2. Or by the lofs of the vital principle itfelf, even when the organization is in a perfect condition, in which cafe the vital organs alone fuffer.

"Death enfues either SUDDENLY, when the body is at once exhaufted of the vital principle; or SLOWLY, which is common natural death.

"Except in thefe fudden cafes, the tranfition from life to death takes place flowly; and the vital principle always lingers in the chief organs, even when it has been withdrawn from all the external parts of the body. In the moft common kind of death, the tranfition takes place by means of apparent death, or apparent external death.

"We ought never to conclude that real death has taken place, until the operation of the chemical powers has begun to produce decompofition and putridity. It is on thefe alone that we can eftablifh any certain figns for afcertaining real death, and not on deficient individual effects of the vital principle, deficient excitability, &c. Unfortunately, therefore, we have no fign of death, on which greater dependance can be put, than corruption, until we fhall be able to difcover the commencement of the free activity of of the chemical powers, which takes place before it is perceptible to us by external figns.

"Every thing that has been faid of the vital principle may be applied to life. Life is a product of the vital principle, depending on the ftate of the organization and external ftimulants. We cannot properly call it, as Brown does, a forced ftate; fince it is as free as the vital principle, though it cannot exift but under certain conditions.

"The different degrees of life are as thofe of the vital principle; Its ftrength or feeblenefs depends on the quantity and activity of the vital principle.

"The quantity and activity of the vital principle are not always in the fame ratio A great deal of life may exift where the activity of the vital principle is fmall, and irritability weak, On the other hand it is poffible that, with a high degree of vital activity, life may be exceedingly feeble. This is immoderate, difeafed vital activity and irritability, which often manifeft themfelves a little before death, and by which the fmall quantity of the vital principle ftill remaining is foon exhaufted. The only proof of a great deal of life is a continual uninterrupted activity of the vital principle, without being exhaufted. Thus people in the years of youth, can, without injury to their powers, undertake very fevere labour.

"For this purpofe a great fufceptibility of the organization for the vital principle is required; and confequently folid, irritable fibres; thefe muft,

"1. Have the property of being eafily acted upon by the vital principle.

"2. Muft be durable enough to bear the continued impulfe of ftimulants.

"The organization lofes both thefe in old age; and hence arife infenfibility, weaknefs, and rigidity."

The fecond part is entitled Afthenocomic, or the means of maintaining and fupporting feeble life; it concludes with the treatment of the fo called incurable difeafes, and the means of palliating urgent fymptoms; fuch as pain, lethargy, heat, thirft, diarrhœa, cramp, &c. &c.

Our readers will readily perceive how much Dr. S. is indebted to the Brunonian Theory, and he does not wifh to conceal it. He fays, "He found fomething good in the work throughout; and he confidered it his duty to make ufe of it."

---

*Practical Obfervations on the Cure of the Gonorrhœa Virulenta in Men.* By Thomas Whately, Member of the Royal College of Surgeons in London. 8vo. pp. 119. Price 2s. 6d. London, 1801, Johnfon, &c.

The Author fupports the generally received opinion refpecting the identity of the Virus of Gonorrhœa and Chancre, in oppofition to Mr. B. Bell, &c. and then proceeds to afcertain the feat of gonorrhœa in men.

"The

" The gonorrhœa virulenta in men was ſuppoſed by many of the old writers, to be a diſeaſed ſecretion from the proſtate gland, the veſiculæ ſeminales, and Cowper's glands; but diſſections by later ſurgeons have demonſtrated, that the ſeat of this diſeaſe is in the urethra, and generally within two inches of its extremity, from which iſſues a diſcharge of a yellowiſh or greeniſh purulent matter. This diſcharge is produced by an inflammation, excited by the action of the venereal poiſon, on the inner membrane of this part of the urethra. The diſeaſe is ſometimes, but not always, attended with a chordee, ardor urinæ, &c. This variation is evidently owing to a difference in the degree of inflammation, and eroſion of the ſurface of the urethra, in ſuch caſes.

" As it will be of conſiderable importance, in the treatment and cure of this complaint, to aſcertain the exact effects produced by the poiſon on the urethra, in all the different varieties of the diſeaſe, I ſhall endeavour to point them out. It has been believed, that the gonorrhœa aroſe moſt frequently from ulcerations in different parts of the urethra, or the glands adjoining to it; but the reſearches of modern anatomiſts ſeem very nearly to have overthrown this opinion, and it has of late been aſſerted, that an ulceration of the urethra never takes place in this diſeaſe. This doctrine appears to have been firſt advanced about the year 1750, by that celebrated anatomiſt the late Dr. Wm. Hunter, who taught it in his lectures. But although it is certainly true, that in moſt inſtances of gonorrhœa there is no ulceration of the urethra, it is equally certain that this is not univerſally the caſe, as ſuch a diſeaſe does ſometimes, though rarely occur.

" In my own practice I have in a common gonorrhœa, now and then, met with ulcers in the urethra. Where its orifice has been naturally wide, I have ſeveral times diſtinctly ſeen them, both a little within, and at the extremity of this canal. In a few other caſes, I have had no doubt, but that ulcers have exiſted in the urethra, within about half an inch of its external orifice, although I have not been able to ſee them. This opinion is founded on the great hardneſs of the part, (far exceeding that which would ariſe from an inflamed mucous gland) on the violence of the inflammation and diſcharge, and on finding the diſeaſe yield to no remedy but mercury given internally, or uſed in frictions. The ulcers in the urethra, which I have been able to inſpect, have been of two kinds; namely, ſuch as were in a painful corroſive or ſpreading ſtate, and attended with an adjoining hardneſs and inflammation; and ſuch as were in an indolent ſtate, with very little hardneſs or inflammation around them. It cannot therefore be doubted, but that the ſame variety exiſts in other caſes, where, from the ſmallneſs of the orifice of the urethra, the fact cannot be aſcertained by inſpection."

Mr. W. ſupports this opinion by quotations from Wiſeman, Aſtruc, Swediaur, Bell, and Monro, and then continues:

" Having, I hope, given ſufficient proofs of the exiſtence of chancres, or ulcers, in the urethra, the above extracts ſhall be followed by a ſhort one, from a very valuable and uſeful publication.

cation.—" Ulcers are alfo feen occafionally in laying open the " urethra, but thefe are not frequent."†

" Where neither of the kind of ulcers before mentioned exifts in the urethra, there is certainly a confiderable difference difcernable, in the depth to which the poifon penetrates into, or excoriates the inner membrane of the urethra, in different cafes of gonorrhœa. Where a chordee and ardor urinæ take place to a confiderable degree, and are attended with an exceffive difcharge, of a greenifh purulent nature, and a confiderable tenfion and inflammation along the courfe of the urethra, the venereal poifon infinuates itfelf deeper into its inner furface, (and particularly into the lacunæ) than in thofe cafes where thefe violent fymptoms do not occur, and where only a moderate yellowifh difcharge takes place. I have frequently perceived this difference by the eye; and I have no doubt of its being always difcernable, were it poffible to fee the whole of the affected furface of the urethra.

" As the fpurious or external gonorrhœa is a difeafe of a very fimilar kind to that which is feated in the urethra, we may, with great propriety, illuftrate by it fome of the varieties of the latter difeafe. Sometimes the gonorrhœa fpuria is attended with large and deep ulcers upon the prepuce or glans, with much inflammation and hardnefs. At other times with thofe which are fmall, and with al efs degree of inflammation and hardnefs. In fome cafes the poifon inflames the tender fkin and fubjacent parts of the glans and prepuce, to fuch a depth, as to thicken and enlarge them; but without producing 'diftinct ulcers. In all thefe affections, a confiderable fecretion of a greenifh or yellow pus takes place. In other cafes, the excoriation of the furface is merely fuperficial, and without any thickening. The inflammation is alfo fo flight as to occafion a fmaller fecretion of pus, of a lighter colour than in any of the other kinds of this difeafe.

" Towards the more complete inveftigation of this fubject, it will be of ufe to obferve, that the gonorrhœa virulenta in general affects the urethra partially; either in irregularly fhaped patches, which are connected with each other, or in diftinct inflamed fpots, which fecrete the purulent matter. I have fometimes been able to fee thefe appearances juft within the orifice of the urethra. Similar appearances are more diftinctly feen in the gonorrhœa fpuria, the thin cuticle on the glans or prepuce being in this cafe eroded by the poifon. At the beginning of the difeafe, when the glans is naturally covered by the prepuce, an erofion of any particular fpot, either on the glans or prepuce, generally produces one, of a correfponding fize and fhape, on the part ufually in contact with it. Sometimes this appearance takes place in a number of diftinct fpots at the fame time; fo that there appear between them irregular portions of found cuticle. If the difeafe in this ftate be neglected, the erofion generally goes on, till the whole of the cuticle on the glans and prepuce is deftroyed; but if the parts be wafhed only with fimple

† Dr. Bailie's Morbid Anatomy.

ſimple water, it is uſually checked in its progreſs. I have no doubt, but that the gonorrhœa vera ſpreads alſo along the inner membrane of the urethra, by the contact of its diſeaſed ſide with the oppoſite ſound ſide; and that it would ſpread in this manner, in a ſhort time, from one end of the urethra to the other, were it not checked by the repeated ablutions of the urine."

Mr. W. in his third chapter, examines the proofs of the diverſity and eſſential difference between the poiſon of gonorrhœa virulenta, and that of the lues venerea, or ſecondary ulcers.

" From what has been advanced, there is reaſon to conclude, that the virus of a chancre, though partaking of the ſame ſpecific property, yet, nevertheleſs, differs conſiderably in its quality from that of an ulcer from a ſecondary infection. Hence we infer, that the poiſon of a bubo is nearly of the ſame kind as that of a chancre; inaſmuch, as being mixed only with a ſmall quantity of lymph in its paſſage to the gland, it has not undergone the ſame change, which it does from a long circulation, and a ſubſequent depoſition from the blood: And experience confirms the truth of this remark; buboes being known to be generally obſtinate and difficult of cure; and when they take place in a perſon afflicted with chancres, they require the ſame quantity of mercury, and the ſame degree of the affection of the mouth, as are neceſſary to cure the chancres.

" If this difference exiſts, between the poiſon of a chancre or a bubo, and that of the lues, we may ſafely conclude, that it exiſts alſo between the poiſon of the gonorrhœa virulenta, and the lues. I ſhall not, therefore, take up my reader's time, in endeavouring to prove this; for if the truth of the former be eſtabliſhed, the latter muſt likewiſe follow; if it be granted, that the poiſon of a gonorrhœa, and that of a chancre, are the ſame. From theſe elucidations of the nature, ſeat, and properties, of the gonorrhœal poiſon, we proceed to the method of treating this complaint.

" From what has been advanced in the preceding pages, it may be ſuppoſed, that the cure of the gonorrhœa virulenta would be found to be ſimple and eaſy; that as mercury, exhibited internally in a proper manner, or uſed in frictions, is known almoſt always to cure a chancre or a bubo, it would, if exhibited in a like manner, cure every ſpecies of this diſeaſe. But in ſuch an expectation we ſhall be diſappointed; for, though mercury, thus introduced into the circulation, will be found, in certain caſes, to have a very conſiderable effect towards removing the diſeaſe, yet it will ſeldom entirely eradicate it, without the aſſiſtance of other remedies.

" I ſhall divide this diſeaſe into three different ſpecies.

" Firſt, The gonorrhœa attended with ulcer in the urethra, or with a conſiderable induration of the lips of its orifice, and of a portion of the corpus ſpongioſum adjoining, but without any apparent ulcer. Theſe appearances being for the moſt part attended with a chordee, and ardor urinæ, to a greater or leſs degree.

" Secondly,

"Secondly, The gonorrhœa attended with a chordee and ardor urinæ, with other marks of a confiderable inflammatory affection of the urethra and penis, but without any appearance of ulcer, or great induration of the lips of the orifice of the urethra, or of the corpus fpongiofum.

"Thirdly, The gonorrhœa unattended with any of thefe circumftances; its chief fymptom being a fmall purulent difcharge from the urethra."

The author then proceeds to lay down the treatment proper for each of thefe fpecies, and their varieties; but our limits do not permit us to enter into this detail, and we muft therefore refer our readers to the work itfelf.

We conclude, though merely from conjecture, that this pamphlet is only a firft part of a complete work on this fevere punifhment of vicious and fenfual indulgences.

---

*A short Account of the Royal Artillery Hospital at Woolwich, with some Observations on the Management of Artillery Soldiers, respecting the Preservation of Health.* By John Rollo, M. D. Surgeon General, Royal Artillery, &c. 8vo. pp. 173, price 5s. London, Mawman.

Our readers muft long have been acquainted with the name and merits of Dr. Rollo, from his Works on Diabetes, and the Ufe of Acids in the Cure of Syphilis. The above correct, ufeful, and philanthropic Compendium of Military Hygieine and Therapeia, does in no refpect derogate from his former fame.

We do moft earneftly recommend thefe Obfervations and Directions to all Officers in the Army, as well as to thofe to whom it is particularly addreffed, in the Royal Regiment of Artillery.

